# Supplementary material for: Online control of the False Discovery Rate in group-sequential platform trials
Source: Stat Methods Med Res. 2022 Oct 3;31(12):2470–85. doi: 10.1177/09622802221129051 (PMC10130539; doi:10.1177/09622802221129051)
Supplement: sj-pdf-1-smm-10.1177_09622802221129051 - Supplemental material for Online control of the False Discovery Rate in group-sequential platform trials [file sj-pdf-1-smm-10.1177_09622802221129051.pdf]

Supplemental material: Online control of the False Discovery Rate in in group-sequential platform trials

Sonja Zehetmayer, Martin Posch, Franz Koenig

July 2022

Contents

1 Abbreviations **2**

2 Order of alternatives **3**

3 Supplemental figures **4**

3.1 Comparison of LOND methods 4

3.2 Direct comparison of CC and NCC+CC controls 9

3.3 OBF versus PO design 11

3.4 Inclusion of additional treatments for stopping in the interim analysis . . . 12

4 Real data example from the RECOVERY platform trial **14**

4.1 Example 1: Analysis of 28-day mortality 14

4.2 Example 2: Receipt of invasive mechanical ventilation or death 16

5 Overview on testing procedures and their FDR control **17**

1 Abbreviations

BH Benjamini-Hochberg

CC concurrent

LOND significance Levels based On Number of Discoveries

LORD significance Levels based On Recent Discovery

FDR False Discovery Rate

FWER Family Wise Error Rat

gs group-sequential

NCC Non-concurrent

NCC+CC Non-concurrent and concurrent

OBF O'Brien-Fleming

PO Pocock

SAFFRON Serial estimate of the Alpha Fraction that is Futilely Rationed On true Null hypotheses

2 Order of alternatives

In the simulations described in the manuscript we considered three different orders of alternative hypotheses (see Fig. 1):

- Random order: In the simulations for each hypothesis the probability for a null hypothesis is π_0. Thus for an individual simulation step, the number of alternatives may differ.
- Alternatives at first: The simulated platform trial starts with $m_1 = K - \pi_0 K$ alternatives followed by $K - m_1$ true null hypotheses.
- Alternatives at last: The simulated platform trials starts with $K - m_1$ true null hypotheses followed by m_1 alternative hypotheses.

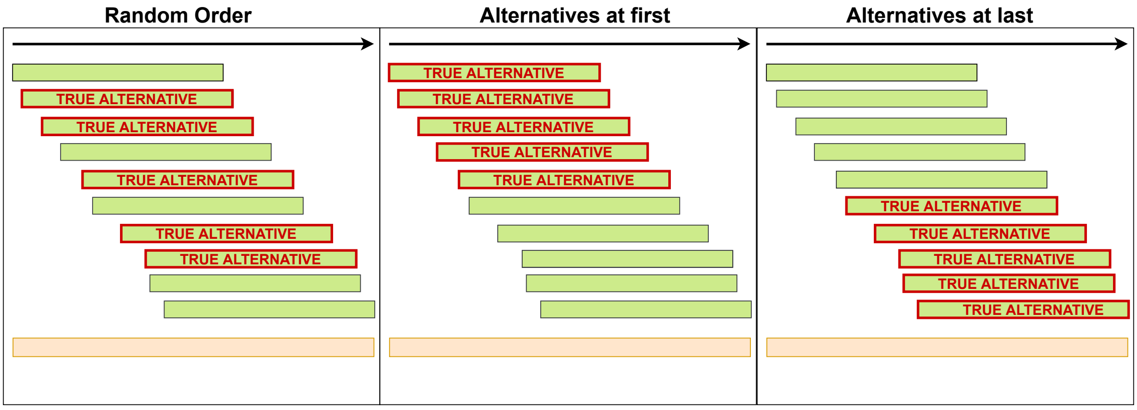

Figure 1: Illustration of different orders of alternative hypotheses for the simulation studies.

3 Supplemental figures

3.1 Comparison of LOND methods

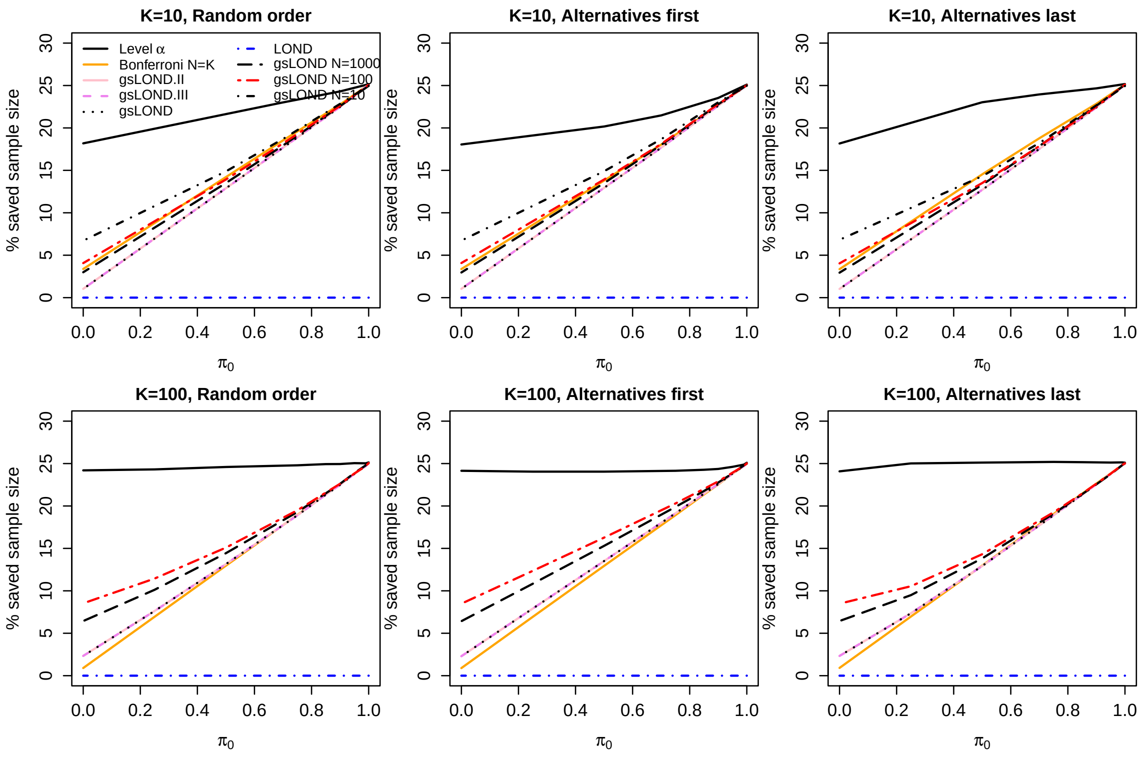

Figure 2: % saved sample size for CC scenario as a function of π_0 for the level-α and the Bonferroni procedure (with $N = K$) and the four LOND procedures LOND, gsLOND, gsLOND.II, and gsLOND.III. O'Brien Fleming design, $N = \{10, 100, 1000, \infty\}$, $\Delta = 0.6$, $\alpha = 0.025$, $\alpha^F = 0.5$. The four LOND procedures can hardly be distinguished as the power values are very similar. Thus, for $N < \infty$, only the power for gsLOND is depicted.

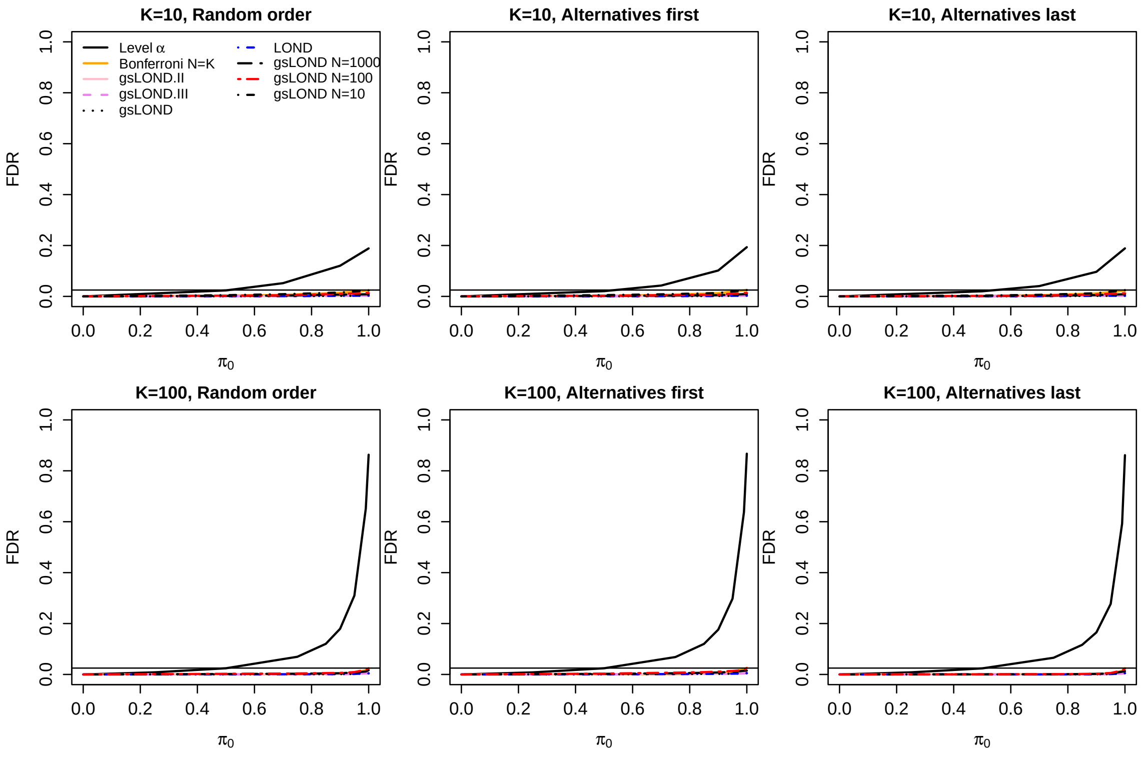

Figure 3: Actual FDR for NCC+CC scenario as a function of π_0 for the level-α and the Bonferroni procedure (with $N = K$) and the four LOND procedures LOND, gsLOND, gsLOND.II, and gsLOND.III. O'Brien Fleming design, $N = \{10, 100, 1000, \infty\}$, $\Delta = 0.6$, $\alpha = 0.025$, $\alpha^F = 0.5$. The four LOND procedures can hardly be distinguished as the power values are very similar. Thus, for $N < \infty$, only the power for gsLOND is depicted.

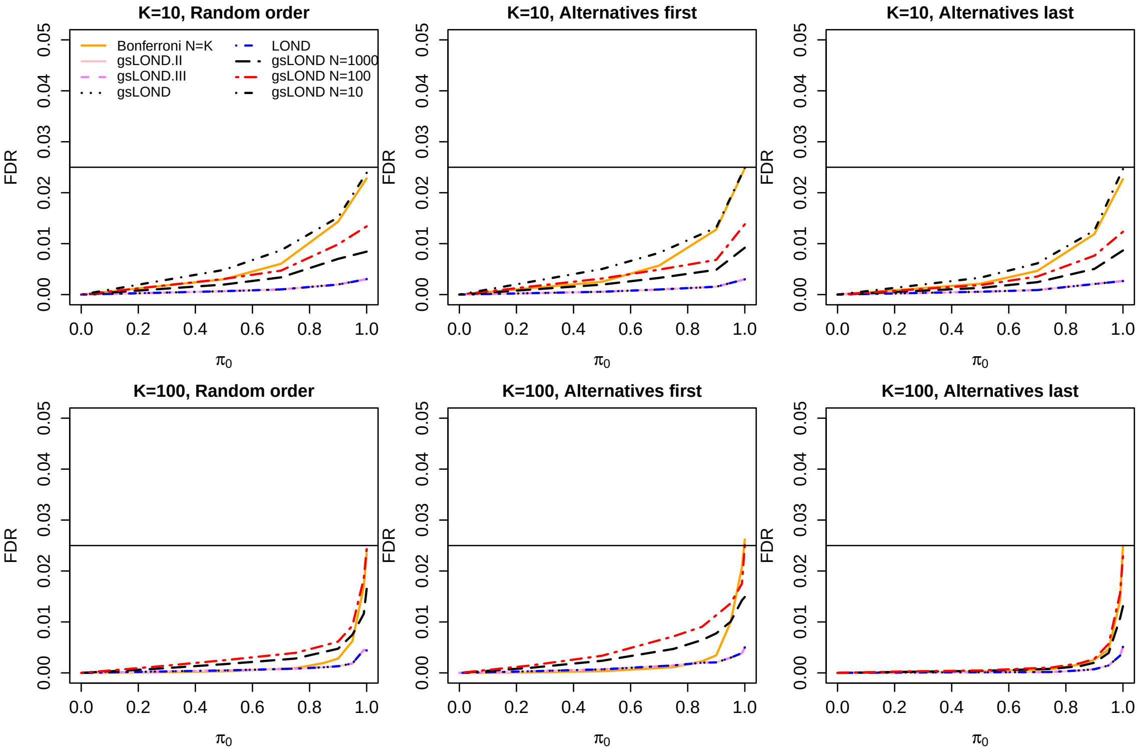

Figure 4: Same as Fig. 3, but with modified y-scales and without the procedure with level α test.

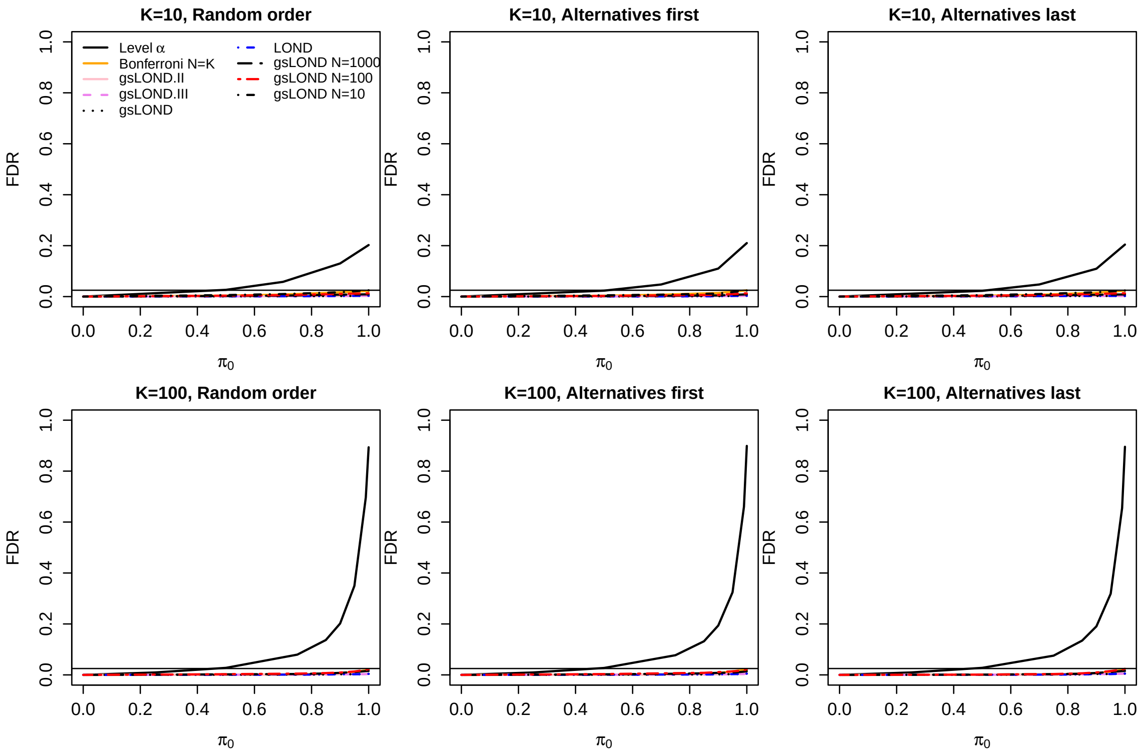

Figure 5: Actual FDR for CC scenario as a function of π_0 for the level-α and the Bonferroni procedure (with $N = K$) and the four LOND procedures LOND, gsLOND, gsLOND.II, and gsLOND.III. O'Brien Fleming design, $N = \{10, 100, 1000, \infty\}$, $\Delta = 0.6$, $\alpha = 0.025$, $\alpha^F = 0.5$. The four LOND procedures can hardly be distinguished as the power values are very similar. Thus, for $N < \infty$, only the power for gsLOND is depicted.

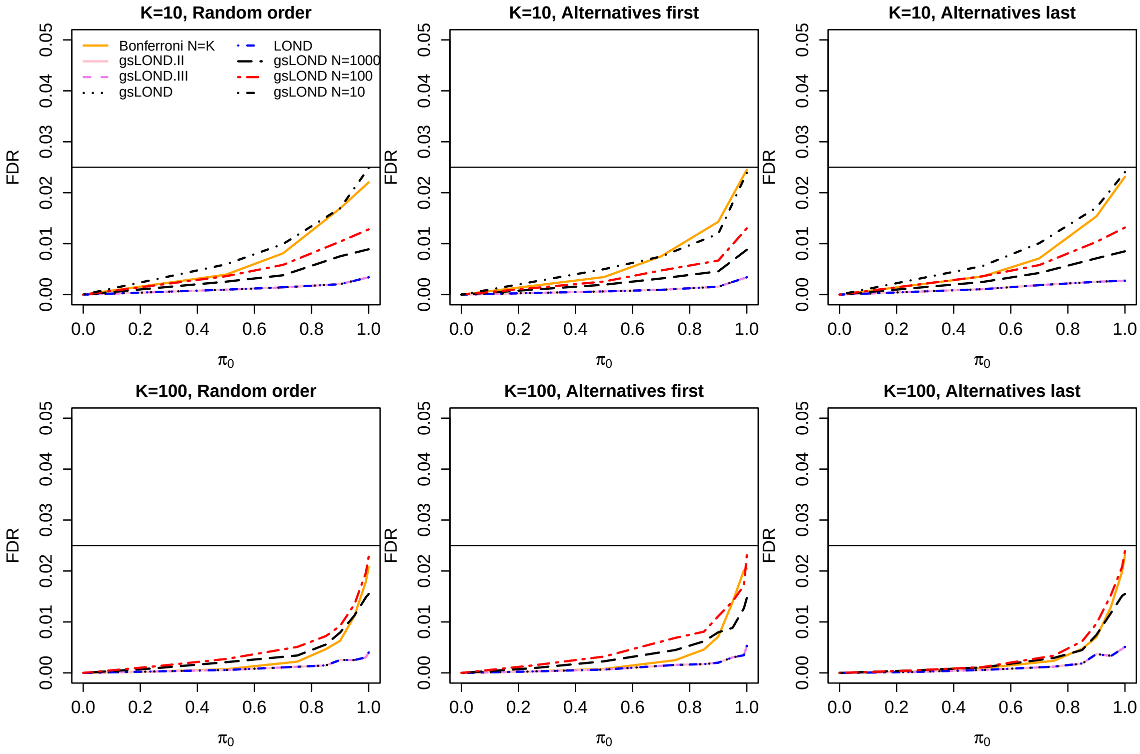

Figure 6: Same as Fig. 5, but with modified y-scales and without the procedure with level α test.

3.2 Direct comparison of CC and NCC+CC controls

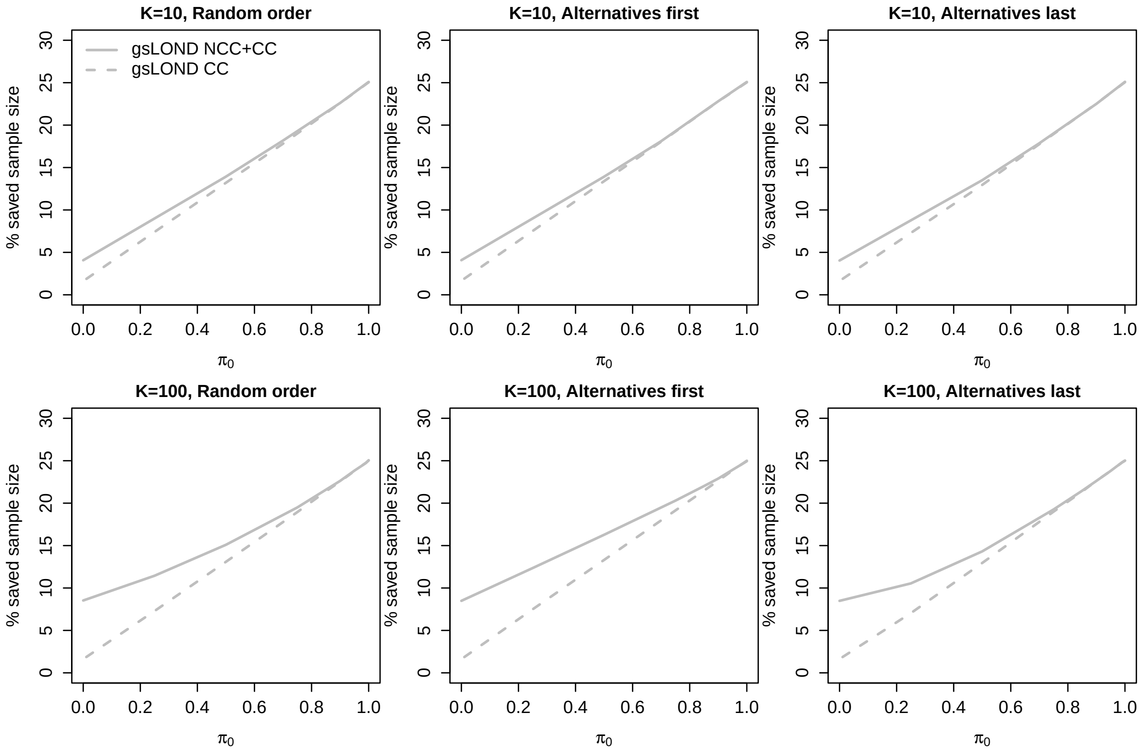

Figure 7: Comparison of % saved sample size for concurrent (CC) versus all (NCC+CC) controls as a function of π_0 for LOND and gsLOND (results for gsLOND.II and gsLOND.III are not depicted due to nearly identical power values); O'Brien Fleming design, $N = 100$, $\Delta = 0.6$, $\alpha = 0.025$, $\alpha^F = 0.5$.

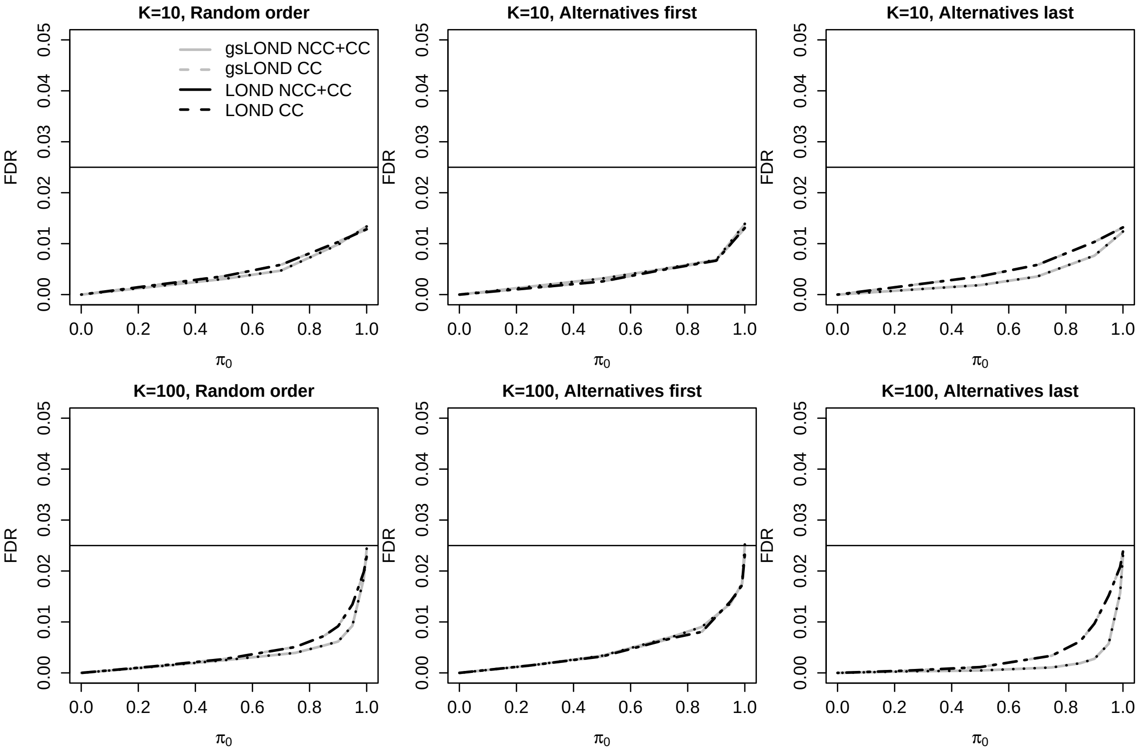

Figure 8: Actual FDR for concurrent (CC) or all (NCC+CC) controls as a function of π_0 for gsLOND.II, and gsLOND.III; O'Brien Fleming design, $N = 100$, $\Delta = 0.6$, $\alpha = 0.025$, $\alpha^F = 0.5$.

Distribution of significance level

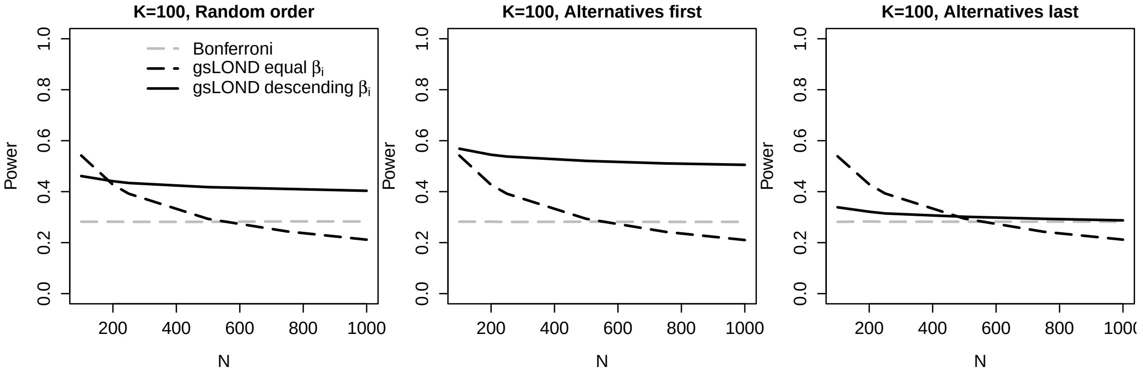

Figure 9: Power comparison of gsLOND (CC controls) for two distributions of significance level as a function of the upper bound N and Bonferroni with $N = K$. The values of β are either derived by a descending or an uniform distribution. OBF design, $\Delta = 0.6$, $\pi_0 = 0.5$, $K = 100$, $\alpha = 0.025$, $\alpha^F = 0.5$.

3.3 OBF versus PO design

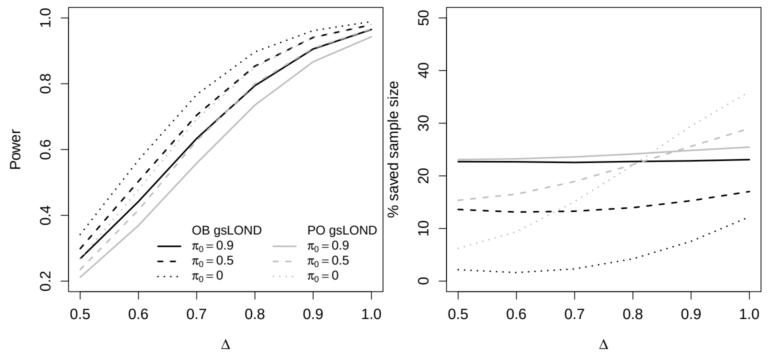

Figure 10: Power values and % saved sample size for OBF and PO designs of gsLOND as a function of the effect size for $\pi_0 = \{0, 0.5, 0.9\}$ and $K = 10$, $N =$, $\alpha = 0.025$, $\alpha^F = 0.5$, CC controls.

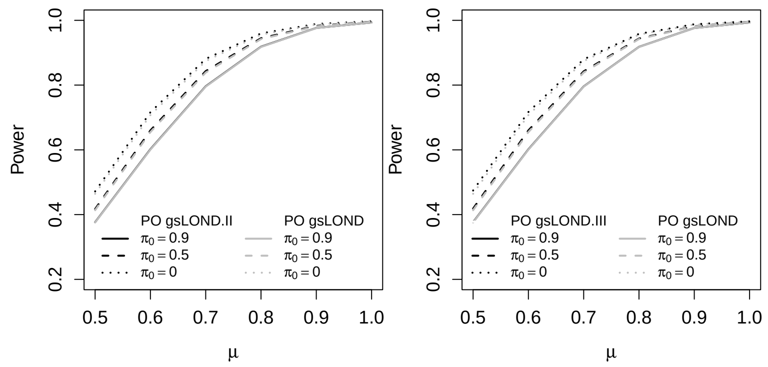

Figure 11: Power values for PO designs of gsLOND and gsLOND.III as a function of the effect size for $\pi_0 = \{0, 0.5, 0.9\}$ and $K = 10$, $N =$, $\alpha = 0.025$, $\alpha^F = 0.5$.

3.4 Inclusion of additional treatments for stopping in the interim analysis

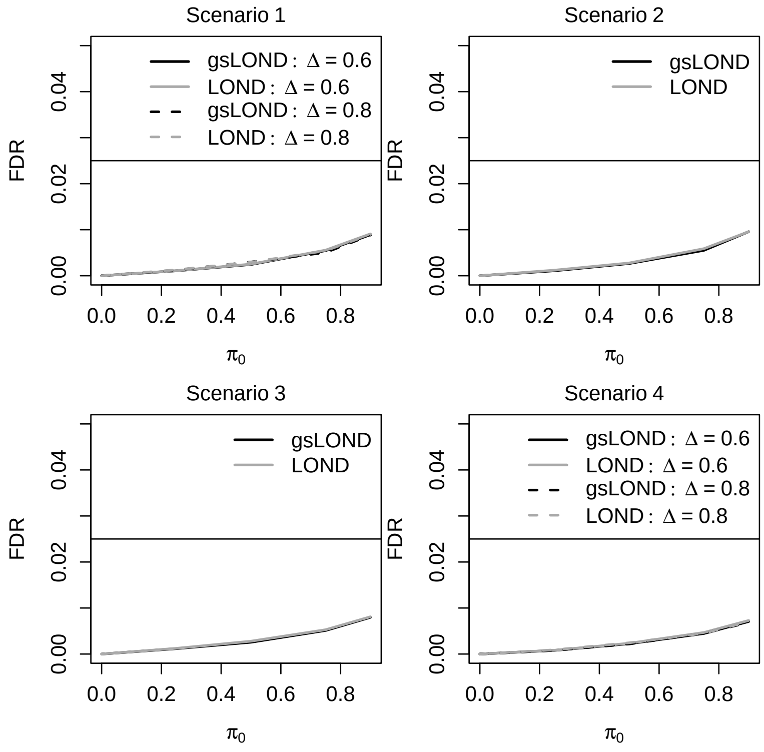

Figure 12: Actual FDR with a fixed budget of the platform trial (NCC+CC) as a function of π_0 for the LOND and gsLOND. If a treatment arm is stopped early, an additional treatment is included. Scenario 1: ratio of true and false alternatives and effect size remains constant, $\Delta = 0.6$ and 0.8. Scenario 2: distributed effect sizes of $\Delta = \{0.4, 0.8, 1.2\}$. Scenario 3: π_0 decreases by 1/80 for each new treatment. Scenario 4: Δ increases to $\Delta = 1$ for additional alternative hypotheses. Initial $K = 10$, $N = 100$, $\alpha = 0.025$, $\alpha^F = 0.5$, OBF design.

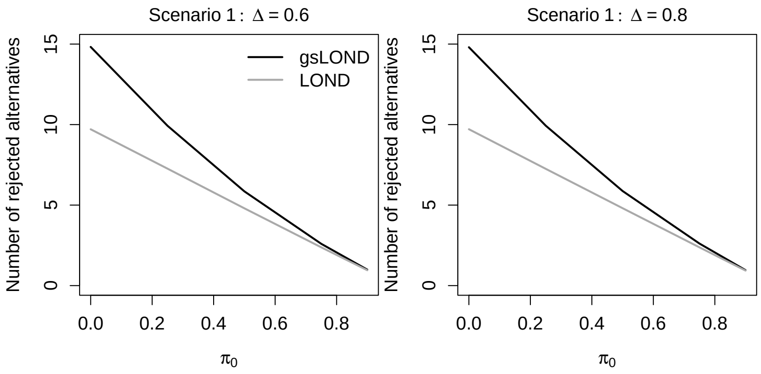

Figure 13: Comparison of number of rejected alternatives with a fixed budget of the platform trial (NCC+CC) as a function of π_0 for the LOND and gsLOND. If a treatment arm is stopped early, an additional treatment is included. Scenario 1: ratio of true and false alternatives and effect size remains constant, $\Delta = 0.6$ and 0.8. Initial $K = 10$, $N = 100$, $\alpha = 0.025$, $\alpha^F = 0.5$, PO design.

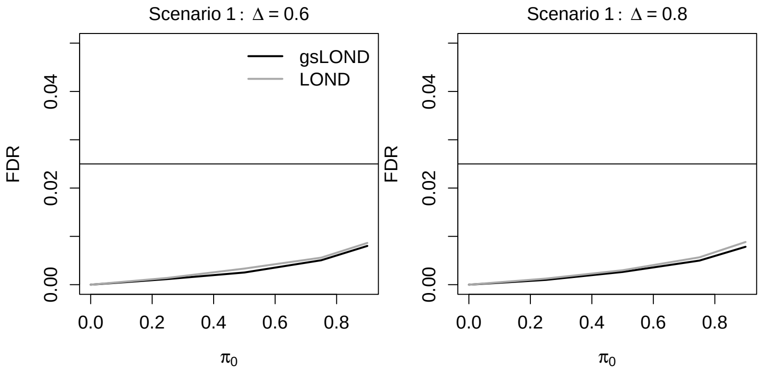

Figure 14: Actual FDR with a fixed budget of the platform trial (NCC+CC) as a function of π_0 for the LOND and gsLOND. If a treatment arm is stopped early, an additional treatment is included. Scenario 1: ratio of true and false alternatives and effect size remains constant, $\Delta = 0.6$ and 0.8. Initial $K = 10$, $N = 100$, $\alpha = 0.025$, $\alpha^F = 0.5$, PO design.

4 Real data example from the RECOVERY platform trial

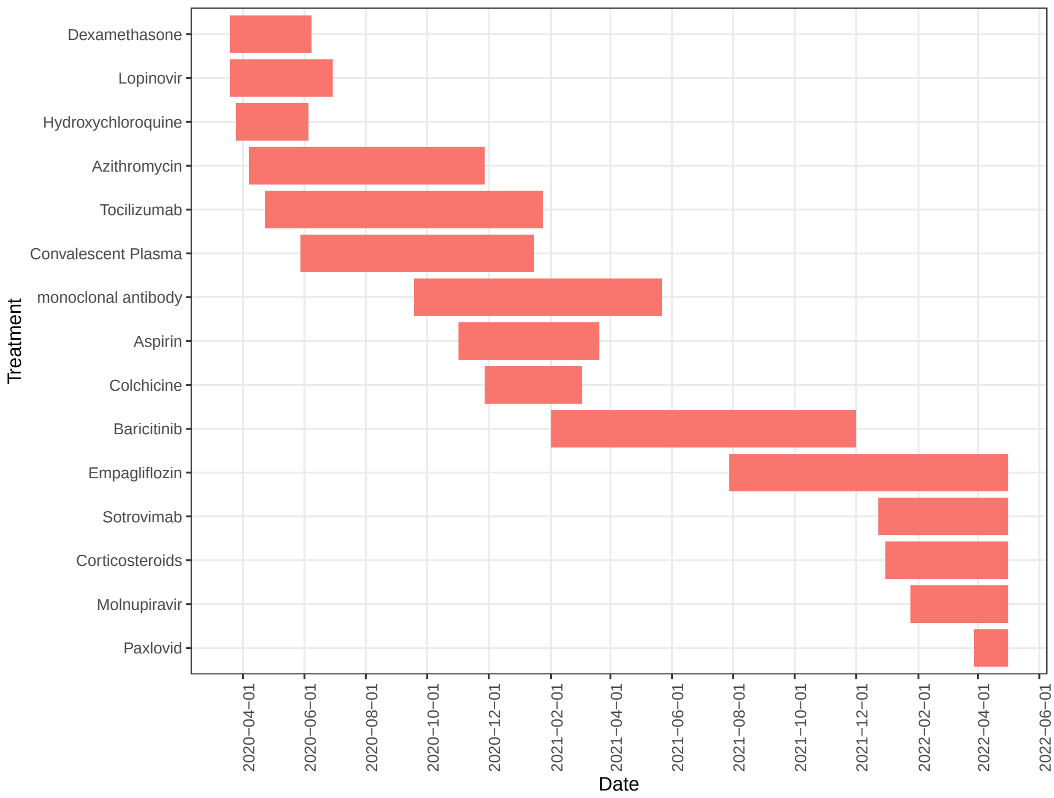

Figure 15: RECOVERY platform trial data: Gantt chart

To reanalyse real data from a platform trial we extracted observations from primary and secondary endpoints from the RECOVERY platform trial from the original manuscripts. We observed the final data of 10 treatments (see Fig. 15, for literature citations of each treatment, see Table 1), 5 treatments were still running at the timing of data extraction.

In the following, two examples are shown using different endpoints for the illustrative calculations. The results presented may differ from the published results as we considered some simplistic assumptions.

4.1 Example 1: Analysis of 28-day mortality

In the original studies, the primary endpoint was 28-day mortality for each treatment. The number of events and the total number of patients for the treatment and the control groups were reported and a time-to-event analysis strategy was applied. In our simplistic reanalysis, we treated the primary endpoint as a binary endpoint and performed chi-square tests for each treatment. We ignored interim analysis or if a trial was stopped in the interim analysis in the original study. We made the hypothetical assumption, that the data as published are the combined results of first and second stage. After observation of half of the published data we assumed that an (hypothetical) interim analysis was performed. Table 1 shows the hypothetical results of stage 1, the corresponding p-values as well as the results of the final analysis.

Table 1: Example 1: 28-day mortality - Hypothetical (stage 1) and observed data (overall).

	Stage 1			Overall		
	Treatment events/total	Control events/total	$p^{(1)}$	Treatment events/total	Control events/total	$p^{(2)}$
Dexamethasone [3]	241/1052	555/2160	0.09	482/2104	1110/4321	0.015
Lopinovir [6]	224/778	436/1640	0.26	449/1556	871/3280	0.07
Hydroxychloroquine [8]	187/808	384/1712	0.69	374/1616	767/3424	0.56
Azithromycin [2]	280/1291	581/2591	0.60	561/2582	1162/5182	0.49
Tocilizumab [11]	310/1011	364/1047	0.047	621/2022	729/2094	0.0051
Convalescent Plasma [9]	700/2898	704/2882	0.81	1399/5795	1408/5763	0.72
monoclonal antibody[7]	198/816	226/760	0.01	396/1633	452/1520	0.00052
Aspirin[5]	611/3676	650/3770	0.48	1222/7351	1299/7541	0.33
Colchicine [10]	586/2805	595/2865	0.91	1173/5610	1190/5730	0.85
Baricitinib [4]	316/2007	335/1946	0.21	631/4014	670/3891	0.09

Table 2: Example 2: Receipt of invasive mechanical ventilation or death - Hypothetical (stage 1) and observed data (overall).

	Stage 1			Overall		
	Treatment events/total	Control events/total	$p^{(1)}$	Treatment events/total	Control events/total	$p^{(2)}$
Dexamethasone	231/890	502/1819	0.37	462/1780	1003/3638	0.21
Lopinovir	224/778	436/1640	0.26	449/1556	871/3280	0.09
Hydroxychloroquine	200/650	352/1312	0.07	399/1300	705/2623	0.01
Azithromycin	302/1215	636/2440	0.43	603/2430	1273/4881	0.24
Tocilizumab	310/877	358/900	0.05	619/1754	715/1800	0.0064
Convalescent Plasma	784/2746	784/2724	0.85	1568/5493	1568/5448	0.79
monoclonal antibody	244/800	272/742	0.0105	488/1599	544/1484	0.0003
Aspirin	736/3496	784/3584	0.40	1473/6993	1569/7169	0.23
Colchicine	672/2671	672/2734	0.62	1344/5342	1343/5469	0.47
Baricitinib	316/2007	335/1946	0.21	631/4014	670/3891	0.07

For the reanalysis we set $N = 20$, $\alpha = 0.05$, and $\alpha^F = 0.5$, OBF design and applied the gsLOND and the LOND procedure. The sequence of hypotheses and the corresponding allocation of the significance level was defined according to the starting times as reported in the literature. As shown in Fig. 15, the duration of the treatments may, however, differ, e.g., treatment monoclonal antibody starts as seventh but stops as ninth treatment.

For the gsLOND as well as the LOND procedure, the treatments dexamethasone and monoclonal antibody were rejected (see table 3). In addition, with gsLOND the treatments Hydroxychloroquine, Azithromycin, Convalescent Plasma, and Colchicine were stopped for futility in the interim analyses and thus a total sample size of up to 808+1291+2898+2805=7802 treatment patients could be saved when ignoring the problem of overrunning (meaning that patients have been already recruited into the platform trial but their outcome data has not been observed when performing the interim analysis). Due to the high recruitment speed, the actual savings would have been much smaller. For

more details see Table 3 showing the results and respective significance boundaries. We assumed that no control patient was saved as other treatments were running in parallel. For $N = 40$ or for gsLOND.II or gsLOND.III, no changes in the results are observed.

4.2 Example 2: Receipt of invasive mechanical ventilation or death

A secondary parameter of the RECOVERY trial is the combined endpoint receipt of invasive mechanical ventilation or death. This is a binary variable where all patients on invasive mechanical ventilation at randomisation are excluded. The numbers are thus smaller compared to the randomized cohort of the primary endpoint. Again we reanalysed the data with Chi-square tests and results might differ from the original analyses where, e.g., additional adjustment factors on age were considered for some treatments. Data are shown in Table 2.

For the reanalysis we set $N = 20$, $\alpha = 0.05$, and $\alpha^F = 0.5$, OBF design, and applied the gsLOND and the LOND procedure. The sequence of hypotheses and the allocation of the significance level was the same as for example 1. The treatment monoclonal antibody was rejected with the gsLOND as well as the LOND procedure (see table 4). In addition, with gsLOND the treatments convalescent plasma and colchicine were stopped for futility in the interim analysis and a total sample size of 2746+2671=5417 could be saved if receipt of invasive mechanical ventilation or death was the primary endpoint. Again no changes in the results were observed for $N = 40$, gsLOND.II or gsLOND.III.

Table 3: Example 1: 28-day mortality - Results and significance boundaries

	R	stopstage	β_i	α_i	$\alpha_i^{(1)}$	$\alpha_i^{(2)}$
Dexamethasone	1	2	0.0189	0.0189	0.000902	0.01861
Lopinovir	0	2	0.0041	0.0082	0.000050	0.00816
Hydroxychloroquine	0	1	0.0035	0.0035	0.000036	NA
Azithromycin	0	1	0.0029	0.0058	0.000096	NA
Tocilizumab	0	2	0.0025	0.0049	0.000070	0.00491
Convalescent Plasma	0	1	0.0021	0.0043	0.000053	NA
monoclonal antibody	1	2	0.0019	0.0038	0.000042	0.00374
Aspirin	0	2	0.0017	0.0034	0.000033	0.00334
Colchicine	0	1	0.0015	0.0030	0.000027	NA
Baricitinib	0	2	0.0014	0.0041	0.000050	0.00411

Table 4: Example 2: Receipt of invasive mechanical ventilation or death - Results and significance boundaries

	R	stopstage	β_i	α_i	$\alpha_i^{(1)}$	$\alpha_i^{(2)}$
Dexamethasone	0	2	0.0189	0.0189	0.000902	0.0186
Lopinovir	0	2	0.0041	0.0041	0.000050	0.0041
Hydroxychloroquine	0	2	0.0035	0.0035	0.000036	0.0035
Azithromycin	0	2	0.0029	0.0029	0.000026	0.0029
Tocilizumab	0	2	0.0025	0.0025	0.000019	0.0025
Convalescent Plasma	0	1	0.0021	0.0021	0.000014	NA
monoclonal antibody	1	2	0.0019	0.0019	0.000011	0.0019
Aspirin	0	2	0.0017	0.0017	0.000009	0.0017
Colchicine	0	1	0.0015	0.0015	0.000007	NA
Baricitinib	0	2	0.0014	0.0028	0.000023	0.0027

5 Overview on testing procedures and their FDR control

In Table 5 we give an overview on the differences between the four LOND procedures with regard to FDR control, nominal significance level, and interim analyses. For the LOND procedure a proof for positively dependent p-values has been given in [12].

For the gsLOND procedures the proof of Zrnic can be used by applying the concept of repeated p-values to address the repeated significance testing in the interim and final analyses. Let $q_i^{(t)}$ denote the repeated p-value for treatment i at stage $t = 1,2$, where we define $q_i^{(2)} = 1$ if the trial stops at the interim analysis (for a definition of the repeated p-value, (see [13], p. 202 or [14]). Then $q_i = \min(q_i^{(1)}, q_i^{(2)})$ are valid p-values of the group-sequential tests (i.e., under the null hypothesis where distribution is larger or equal compared to the uniform distribution on $[0,1]$). Therefore, assuming independence of the q_i, the (updated) LOND procedure, applied to the q_i controls the FDR.

Now, the group-sequential LOND procedure (gsLOND and gsLOND.II) is strictly more conservative than the above procedure, because it can be written as a LOND procedure, where for some hypotheses the p-value $q_i^{(1)} (\geq q_i)$ is chosen for the testing. The argument does not directly apply for the positively dependent case, as it would have to be shown that the q_i satisfy the positive regression dependency on a subset (PRDS). For the gsLOND this should be the case because for non-overlapping treatment arms the p-values are independent when using CC control data only and the others should be positively correlated due to the shared CC control data.

Table 5: Comparison of the four LOND procedures.

	Design	Significance level	Interim analysis	FDR control
LOND	Fixed sample design	Full nominal significance level α_i dedicated to a specific hypothesis H_i can be spend at the final single analysis	No interim analysis	Proof for updated LOND for positively dependent p-values [12]
gsLOND	Group-sequential design	Nominal significance level dedicated to a specific H_i can differ compared to LOND due to interim analyses of testing H_j $j \leq i$ (e.g., already higher nominal significance level α_i due to early rejections of H_j, $j < i$. Uses same spending function in case that the nominal level α_i is increased after the interim analysis.	Interim analysis for early stopping for efficacy or futility, smaller average sample sizes due to early stopping. Requires increase of maximum sample size to keep same power as LOND.	Proof sketch available assuming independence. For positive correlations only shown by simulations for several scenarios.
gsLOND.II	Group-sequential design: update and recalculate α_i to exhaust level	Exhaust level α when updating nominal significance level α_i between interim and final analysis. Therefore potentially more rejections compared to gsLOND in the final analysis. Calculation of critical boundaries becomes more complex.	Interim analysis for early stopping for efficacy or futility. same average sample size as gsLOND but higher power	FDR control as for gsLOND
gsLOND.III	Group-sequential design: update α_i in case of any in-between rejections	After rejection of H_i, update of nominal significance level α_j for all hypotheses H_j without final analysis ($j < i$ or $j > i$). Potentially higher number of rejections.	Interim analysis for early stopping for efficacy or futility. Average sample size similar or smaller compared to gsLOND depending on timing of interim analyses	Update of nominal significance level not in line with prefixed order as defined for the original LOND procedure. No proof for FDR control, potentially higher FDR than gsLOND; In simulations no inflation was observed.

References

[1] DeMets, D. L. and Lan, K. K. (1994). Interim analysis: the alpha spending function approach. Stat. Med., 13(13-14):1341–52; discussion 1353–6.

[2] RECOVERY Collaborative Group (2021). Azithromycin in patients admitted to hospital with COVID-19 (RECOVERY): a randomised, controlled, open-label, platform trial. Lancet, 397: 605–12.

[3] RECOVERY Collaborative Group (2021) Dexamethasone in Hospitalized Patients with Covid-19. N Engl J Med 2021;384:693-704

[4] RECOVERY Collaborative Group (2022) Baricitinib in patients admitted to hospital with 3 COVID-19 (RECOVERY): a randomised, controlled, 4 open-label, platform trial and updated meta-analysis. medRxiv preprint https://doi.org/10.1101/2022.03.02.22271623

[5] RECOVERY Collaborative Group (2020)Aspirin in patients admitted to hospital with COVID-19 (RECOVERY): a randomised, controlled, open-label, platform trial. Lancet 2022; 399: 143–51

[6] RECOVERY Collaborative Group (2020) Lopinavir–ritonavir in patients admitted to hospital with COVID-19 (RECOVERY): a randomised, controlled, open-label, platform trial. Lancet 2020; 396: 1345–52

[7] RECOVERY Collaborative Group (2020) Casirivimab and imdevimab in patients admitted to hospital with COVID-19 (RECOVERY): a randomised, controlled, open-label, platform trial. Lancet 2022; 399: 665–76

[8] RECOVERY Collaborative Group (2020) Effect of Hydroxychloroquine in Hospitalized Patients with Covid-19. N Engl J Med 383;21

[9] RECOVERY Collaborative Group (2021) Convalescent plasma in patients admitted to hospital with COVID-19 (RECOVERY): a randomised controlled, open-label, platform trial. Lancet 2021; 397: 2049–59

[10] RECOVERY Collaborative Group (2021) Colchicine in patients admitted to hospital with COVID-19 (RECOVERY): a randomised, controlled, open-label, platform trial. Lancet Respir Med 2021; 9: 1419–26

[11] RECOVERY Collaborative Group (2021) Tocilizumab in patients admitted to hospital with COVID-19 (RECOVERY): a randomised, controlled, open-label, platform trial. Lancet 2021; 397: 1637–45

[12] Zrnic,T. and Ramdas, A. and Jordan, M.I. (2021) Asynchronous online testing of multiple hypotheses. J. Mach. Learn. Res.; 22: 1-39.

[13] JENNISON, C. and TURNBULL, B. W. (2000). Group Sequential Methods with Applications to Clinical Trials. Boca Raton, FL: Chapman and Hall/CRC.

[14] Posch, M., Wassmer, G., and Brannath, W. (2008). A note on repeated p-values for group sequential designs. Biometrika, 95(1), 253-256.
